# Stability of Monolithic MOF Thin Films in Acidic and Alkaline Aqueous Media

**DOI:** 10.3390/membranes11030207

**Published:** 2021-03-15

**Authors:** Tawheed Hashem, Elvia P. Valadez Sanchez, Evgenia Bogdanova, Anna Ugodchikova, Alaa Mohamed, Matthias Schwotzer, Mohamed H. Alkordi, Christof Wöll

**Affiliations:** 1 Institute of Functional Interfaces (IFG), Karlsruhe Institute of Technology (KIT), Hermann-von Helmholtz-Platz 1, 76344 Eggenstein-Leopoldshafen, Germany; valadez.elvia@gmail.com (E.P.V.S.); alaa.khalil2@partner.kit.edu (A.M.); matthias.schwotzer@kit.edu (M.S.); christof.woell@kit.edu (C.W.); 2 School of Nuclear Science & Engineering, National Research Tomsk Polytechnic University, Lenina Avenue 30, 634050 Tomsk, Russia; evgeniyabog@mail.ru (E.B.); Avu10@tpu.ru (A.U.); 3 Center for Materials Science, Zewail City of Science and Technology, October Gardens, 6th of October, 12578 Giza, Egypt; malkordi@zewailcity.edu.eg

**Keywords:** MOF, SURMOF, liquid phase epitaxy, layer-by-layer, pH stability

## Abstract

In the context of thin film nanotechnologies, metal-organic frameworks (MOFs) are currently intensively explored in the context of both, novel applications and as alternatives to existing materials. When it comes to applications under relatively harsh conditions, in several cases it has been noticed that the stability of MOF thin films deviates from the corresponding standard, powdery form of MOFs. Here, we subjected SURMOFs, surface-anchored MOF thin films, fabricated using layer-by layer methods, to a thorough characterization after exposure to different harsh aqueous environments. The stability of three prototypal SURMOFs, HKUST-1, ZIF-8, and UiO-66-NH_2_ was systematically investigated in acidic, neutral, and basic environments using X-ray diffraction and electron microscopy. While HKUST-1 films were rather unstable in aqueous media, ZIF-8 SURMOFs were preserved in alkaline environments when exposed for short periods of time, but in apparent contrast to results reported in the literature for the corresponding bulk powders- not stable in neutral and acidic environments. UiO-66-NH_2_ SURMOFs were found to be stable over a large window of pH values.

## 1. Introduction

Over the last few years, there has been an increasing demand for sustainable alternatives for current and new products and processes. In this context, thin film nanotechnologies play a particularly important role. Although great progress has been achieved [1,2,3], many open questions including the understanding, development, and application of nanostructures remain open. Also, limitations of existing materials have created a quest for new material platforms. With regard to the latter, metal organic frameworks (MOFs), a class of highly crystalline and porous materials, have lately attracted immense attention. The hybrid and modular nature of these materials which are assembled by connecting metal or metal-oxo nodes through multitopic organic linkers has permitted the formation of over 70,000 different reported frameworks [4]. MOFs show a large variety of topologies, window apertures, pore sizes, and functionalities. Given such versatility, the application of MOFs has been explored in multiple fields including gas storage, gas- and liquid-phase separation, sensor technologies, electrochemistry, among others [5,6,7,8,9]. Likewise, the study and production of high-quality thin MOF films supported on flat substrates has been evolving quickly and continues to be an important point of research with great areas of opportunity [10,11,12]. 

A particularly interesting example of high-quality MOF thin films are surface-supported metal-organic frameworks (SURMOFs). These high-quality monolithic thin films can be grown using layer-by-layer methods [13]. Previous works have shown that high-quality, crack/pinhole-free homogeneous MOF thin films can be obtained by careful optimization of the concentration, immersion times, and temperatures of the reactant solutions. For a growing number of MOF materials, appropriate layer by layer (LBL) synthesis conditions yielding high-quality SURMOFs have been developed, including HKUST-1 [14], ZIF-8 [15], and UiO-66-NH_2_ [16]. Such MOF thin films have been characterized using a wide range of techniques and have been also successfully used to fabricate devices for diverse applications delivering high-performance results [13]. In particular, for optical applications these MOF thin films have superior properties and outperform layers which are formed e.g., by coating substrates with suspensions made from the powdery form of MOFs obtained using conventional, solvothermal synthesis schemes [17]. 

When it comes to the application of MOFs within the fields of catalysis [18], electrocatalysis [19], battery separators [20], water desalination process [21], and biological applications [22,23], determining the stability of SURMOF films in an aqueous environments is critical. By exploring the behavior of different SURMOF films under relatively harsh environments, a better understanding of their potential and limitations can be obtained. 

In this work, HKUST-1 (also called MOF-199 and Cu-BTC), ZIF-8, and UiO-66-NH_2_ SURMOF films were exposed to various acidic and alkaline environments at room temperature over different periods of time. Their crystallinity was inspected after each step in order to capture any changes that affect their structural integrity. Scanning electron microscope (SEM) images were also collected and presented in the following sections. The results were also compared and contrasted to published works studying these MOF materials in their bulk form, powders. This stepwise study permitted to obtain a more detailed characterization of diverse SURMOF films under specific conditions. As suggested by Liu et al. [13], such information is mandatory when selecting among different available MOF and SURMOF synthesis methods with regard to specific applications.

## 2. Experimental Section

### 2.1. Preparation of HKUST-1 Surface-Anchored Metal-Organic Framework (SURMOF) Films

Au-coated silicon wafers were functionalized by deposition of self-assembled monolayers (SAMs) using a 20 µM concentration of ethanolic solution of 16-mercaptohexadecanoic acid (MHDA, Sigma Aldrich, Darmstadt, Germany). The wafers were left in the solution for 72 h at room temperature in order to obtain a –COOH functionalized surface. As a final step, the substrates were rinsed with pure EtOH and dried with nitrogen for their immediate use.

The HKUST-1 SURMOF films were then synthesized using the LBL liquid phase epitaxial (LPE) hand spraying method. First, the substrate was placed on a holder and alternatively sprayed using the following 6 step, 2 min approach: (1) Spray for 15 s using a 1 mM ethanolic solution of Cu(OAc)_2_ (Merck, Darmstadt, Germany), (2) Wait for 35 s, (3) Rinse for 5 s using pure EtOH, (4) Spray for 25 s using a 0.2 mM ethanolic solution of 1,3,5-benzenetricarboxylic acid (Alfa Aesar, Kandel, Germany), (5) Wait for 35 s, and (6) Rinse again with pure EtOH for 5 s. This procedure was then repeated 50 times. 

### 2.2. Preparation of ZIF-8 SURMOF Films 

Prior to the deposition process, Au-coated silicon wafers were immersed in a 1 mM ethanolic solution of 11-mercapto-1-undecanol (MUD, Sigma Aldrich, Darmstadt, Germany). The substrates were left in the solution for 24 h at room temperature in order to coat the Au substrate with a self-assembled monolayer (SAM) exposing an OH-terminated organic surface. As a final step, the substrates were rinsed with pure EtOH and dried with nitrogen for their immediate use.

On these functionalized substrates, the ZIF-8 SURMOF films were deposited using the LBL LPE dipping method [15]. The substrates were placed in a MSM Robust carousel tissue stainer (Slee Thomas-Medical, Mainz, Germany) using a sample holder. A 4-step dipping program was used for the deposition process. First, the substrates were dipped for 300 s in a 10 mM methanolic solution of zinc nitrate hexahydrate 98% (Alfa Aesar, Kandel, Germany). Next, the substrates were immersed for 100 s in MeOH as a rinsing step. Third, they were dipped for 300 s in a 20 mM methanolic solution of 2-methylimidazole (Merck, Darmstadt, Germany), and finally rinsed again for 100 s in MeOH completing 1 cycle. Both solutions and rinsing steps were prepared using methanol SeccoSolv^®^ max. 0.003% H_2_O (Merck, Darmstadt, Germany). The 4-step process was then repeated to complete 100 cycles. 

### 2.3. Preparation of UiO-66-NH_2_ SURMOF Films

UiO-66-NH_2_ SURMOF samples were synthesized using the previously reported method [16]. First, Au-coated silicon wafers were functionalized using a 1 mM ethanolic solution of 11-Mercapto-1-undecanol (MUD, Sigma Aldrich, Darmstadt, Germany). The substrates were kept in the dark immersed into this solution for 24 h at room temperature, resulting in an -OH functionalized surface. Finally, the substrates were thoroughly rinsed with absolute EtOH and dried under nitrogen flow for their immediate use.

Next, the LBL LPE manual dip coating method was used for the UiO-66-NH_2_ SURMOF samples. The metal ion source solution contained 90 mM of ZrCl_4_ (Alfa Aesar, Kandel, Germany) dissolved in 10 mL *N*,*N*-dimethylformamide (DMF), (Emsure Merck, Darmstadt, Germany) + 2 mL hydrochloric acid 37% (HCl), (VWR Chemicals, Radnor, PA, USA). The organic linker source solution contained 150 mM of 2-aminoterepthalic acid (Alfa Aesar, Kandel, Germany) dissolved in 10 mL DMF. These two reactant solutions were heated up to and maintained at 70 °C during all the preparation steps. Each deposition cycle started with an immersion of the functionalized substrates into the metal ion source solution under constant stirring of the solution at 500 rpm for 90 min. Then, the substrates were rinsed with DMF for 5 min. Next, the substrates were immersed in the organic linker source solution under constant stirring at 500 rpm for 120 min. The deposition cycle was completed by rinsing the substrates again with DMF for 5 min. This 4-step process was repeated 30 times yielding a 30 cycle UiO-66-NH_2_ SURMOF supported on the functionalized substrates. The stirring of the solution was necessary in order to obtain homogeneous nucleation and a continuous coating of the SURMOF atop the substrate [24]. The resulting samples were then rinsed with pure EtOH 6 times and then left in pure EtOH for 24 h in order to ensure a complete exchange of guest molecules. Finally, the samples were removed and dried in air overnight. 

### 2.4. Characterization Techniques

X-ray diffraction (XRD) characterization was performed using a Bruker D8-Advance “DaVinci” (Ettlingen, Germany) in Bragg-Brentano θ-θ geometry and a 192-stripe Lynxeye detector over an angular range of 2θ from 3° to 15° with a 0.018–0.025° 2θ step width and 384 seconds per step for all SURMOFs samples (HKUST-1, ZIF-8 and UiO-66-NH_2_).

Scanning electron microscopy (SEM) was used to investigate the morphologies of the films. SEM measurements were conducted using a FEI Philips XL 30 field-emission gun environmental scanning electron microscope (FEG-ESEM) (FEI Co., Hillsboro, OR, USA). In order to avoid charging and increase sample conductivity, all samples were coated with a thin layer (3–5 nm thick) gold/palladium film. The measurements were taken under a high vacuum (1.5 Torr) using 20 keV acceleration voltage.

Infrared reflection absorption spectroscopy (IRRAS) data was obtained under ambient conditions with a VERTEX 80 FTIR (Fourier transform infrared) spectrometer (Bruker, Ettlingen, Germany) equipped with an accessory (A518) providing a fixed angle of incidence of 80°, and a liquid-nitrogen-cooled mercury cadmium telluride (MCT) narrow band detector. Perdeuterated hexadecanethiol-SAMs on Au/Ti/Si were used for reference measurements. Please refer to Figure S1 (Supplementary Material) for the IRRAS spectra of the studied films in this work. Here, the initial composition of the films could be confirmed, matching the characteristic bands of each material [14,25,26,27,28,29,30].

### 2.5. Chemical Stability Experiments in Acidic, Neutral, and Alkaline Media

After synthesis, the HKUST-1, ZIF-8, and UiO-66-NH_2_ samples were characterized with XRD, IRRAS, and SEM in order to confirm their crystallinity, composition, and morphology, respectively. 

In order to assess the stability of the HKUST-1, ZIF-8, and UiO-66-NH_2_ SURMOF films in different and contrasting environments, the SURMOFs were immersed into solutions with different pH values (HKUST-1: pH = 2, 7, and 11, ZIF-8: pH = 2, 4, 7, and 11, and UiO-66-NH_2_: pH = 2, 7, 10, and 11). The pH of the different solutions was adjusted with distilled water using 1 M HCl (Merck, Darmstadt, Germany, analytical grade) for the acidic environments and 1 M NaOH (Merck, Germany, 99% purity) for the basic environments.

Prior to immersion of the samples into the different solutions, each individual sample was divided into two pieces (1 cm × 1 cm). Each piece of the HKUST-1, ZIF-8, and UiO-66-NH_2_ SURMOF samples was treated in a given pH condition at room temperature in the following way. The sample was first immersed in the media (e.g., pH 2) for 1 min. Then rinsed 3 times with EtOH and finally dried in a stream of dry nitrogen. Next, it was characterized with XRD in order to observe any changes of the sample’s crystallinity. Depending on the result, the same sample was then immersed for longer durations (5 min, 10 min, 20 min, 60 min, and 120 min.) and likewise rinsed and characterized with XRD using same diffractogram collection time and parameters. As a last step for each of the samples, SEM images were recorded. It is important to mention that the pH values of the solutions were also measured after immersion. This was done in order to verify that the pH of the solution was not subject to change once in contact with the sample.

## 3. Results and Discussion

### 3.1. HKUST-1 SURMOF Films

**Figure 1:**
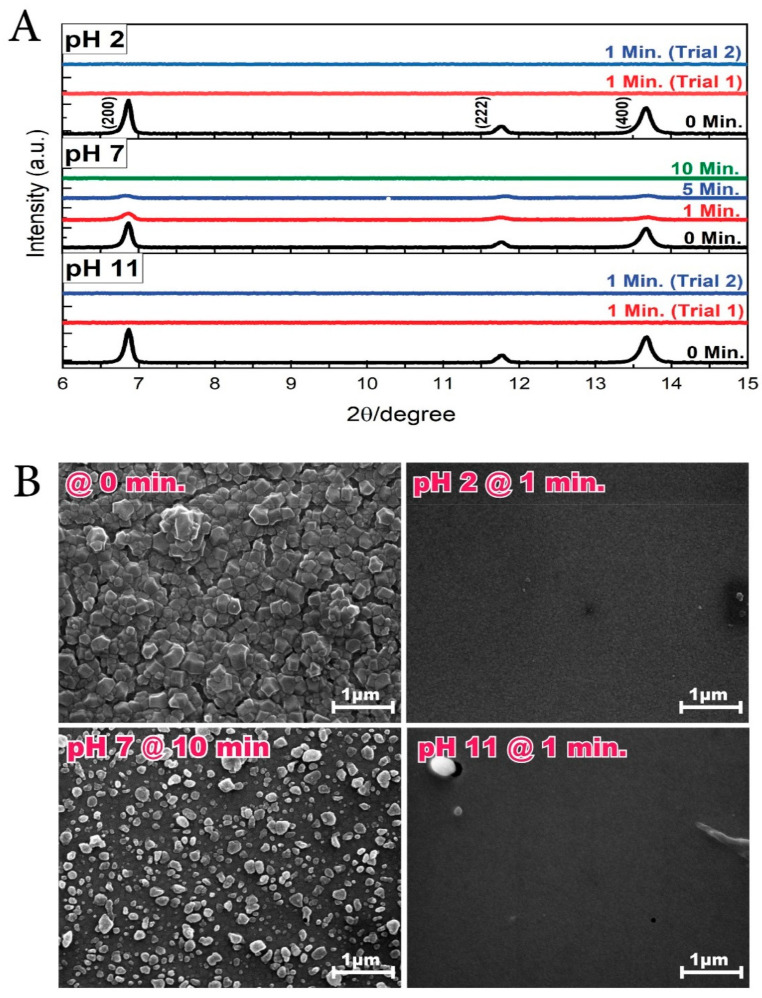
(**A**) X-ray diffraction (XRD) patterns and (**B**) scanning electron microscopy (SEM) images of HKUST-1 SURMOF films immersed in pH 2, pH 7, and pH 11 solutions.

The HKUST-1 films were immersed in acidic (pH 2) and alkaline (pH 11) environments. The XRD patterns recorded for each scenario are reported in Figure 1A. As it can be observed, neither scenario resulted in stable films, even after just 1 min of immersion. The crystallinity of the material was completely destroyed and the HKUST-1 material itself appears to have been completely delaminated from the surface, as shown in the SEM images obtained (Figure 1B). Experiments were repeated to ensure the reliability of the information recorded, but the same results were obtained (Figure 1A, Trial 2). Concerning a pH 7 environment (Figure 1A), the films also demonstrated poor stability, losing completely their crystallinity after 10 min. Nonetheless, the SEM images demonstrated that the film was not completely removed from the substrate but rather transformed into an amorphous material. 

Several previous works have reported that the prolonged exposure of HKUST-1 powder in water/moisture leads to a fast degradation of this crystalline material [31,32,33]. Additional efforts have been also conducted in order to improve the mechanical, thermal, and moisture stability of HKUST-1 by using hybrid or composite materials and mixed matrix membranes, further enhancing the material’s properties [34,35,36]. Nonetheless, given the almost immediate decomposition in aqueous environments observed in these experiments, using HKUST-1 MOF thin films with this or larger thicknesses does not appear promising for applications in any aqueous environment. For such applications, HKUST-1 is clearly not the material of choice and other types of SURMOF are more appropriate for application under such conditions. Interestingly, in a recent investigation, the intrinsic structural instability of HKUST-1 in an aqueous environment was attributed to a cascade of reactions initiated by Cu-coordinated water molecules that can undergo spontaneous deprotonation and formation of Cu-OH species, eventually leading to a liberation of the organic linkers and the deconstruction of the molecular framework [37]. In a related study, Müller et al. [38] pointed out the possibility of healing defects in HKUST-1 SURMOF films resulting from exposure to water vapor by exposing the samples to its synthesis solvent, ethanol. 

### 3.2. ZIF-8 SURMOF Films

**Figure 2:**
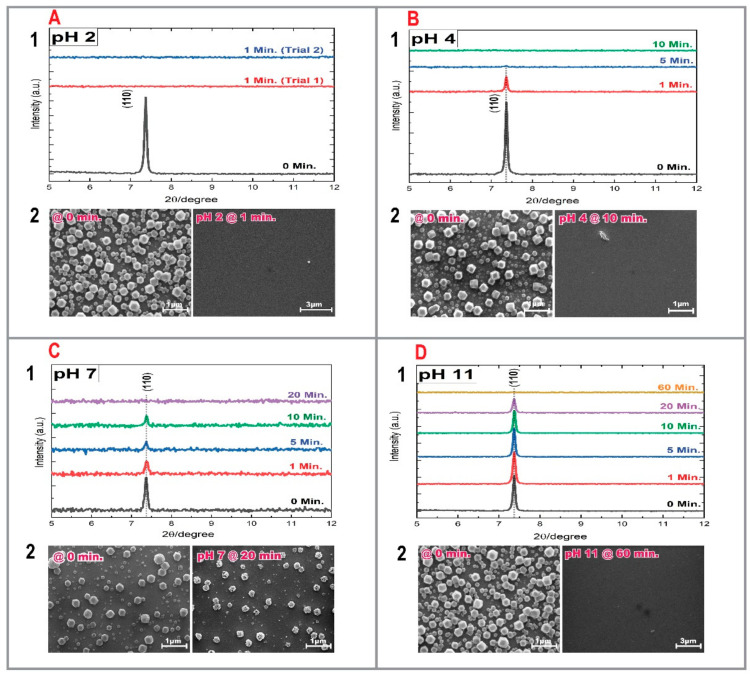
XRD patterns and SEM images of ZIF-8 SURMOF films immersed in a: (**A**) pH 2 solution, (**B**) pH 4 solution, (**C**) pH 7 solution, and (**D**) pH 11 solution.

When exposed to a highly acidic environment (pH 2), the crystallinity of the ZIF-8 SURMOF film was lost already after very short immersion times (1 min); see XRD and SEM data reproduced in Figure 2A. As the environment was turned less acidic (pH 4), the sample started showing slight changes regarding its stability. The characteristic (110) reflex was still present after 1 min, substantially reduced after 5 min, and completely lost after 10 min (cf. Figure 2B). Under neutral conditions (pH 7), ZIF-8 SURMOF films revealed only a slight improvement in stability. The characteristic (110) reflex was still present after 10 min and completely lost after 20 min. In contrast to the pH 2 and pH 4 scenarios, the film was not completely detached from the surface, but was indeed transformed into an amorphous material (cf. Figure 2C). Finally, exposure of ZIF-8 SURMOF films in a basic environment (pH 11) resulted in a more pronounced stability. As can be seen from the XRD data reproduced in Figure 2D, the crystallinity of the film remained high for immersion times of up to 20 min. Overall, the intensity and half-width of the characteristic (110) reflex showed no substantial variation. Nonetheless, after an immersion time of 60 min, the XRD-data revealed that the crystallinity of the film was lost. According to the SEM images, the film had been removed completely from the substrate. Overall, all of these results agree with the known excellent stability of low valency metal ions and azolate-based ligands in alkaline media and its poor stability in acidic environments [39].

Despite a relatively large number of previous investigations aimed at exploring the stability of the ZIF-8 bulk material in various conditions, not much has been reported about thin films. Such information, however, is of crucial importance for the application of thin films. Concerning the stability of ZIF-8 in water/moisture environments, various outcomes have been reported [40,41,42,43,44]. Zhu et al. [40] and Duke et al. [41] studied the stability of ZIF-8 in seawater. The former reported a highly stable material, while the latter observed a 1% loss of the total mass of the original ZIF-8, although the crystallinity was preserved. In the case of this work, at pH 7 the ZIF-8 SURMOF film was turned into an amorphous material after 20 min immersion. Furthermore, Pan et al. [42] explored a pure aqueous synthesis route for ZIF-8 and reported excelling stability of the material in boiling water and methanol. Considering ZIF-8 SURMOFs like the ones explored in this work, a previous work [16] showed that the films lost their crystallinity after being immersed in boiling water for 1 h and SEM images revealed a complete detachment of the film from the substrate. Moreover, Sheng et al. [44] explored the effect of varying Zn salts on the hydrothermal stability of ZIF-8 and observed significant degradation in the material after 10 days with the exception of the ZIF-8 derived from Zn acetate. Considering various contrasting results, Zhang et al. [45] tested ZIF-8 powders obtained via different synthesis routes, which resulted in different crystallite sizes. From the results of their experiments, Zhang et al. [45] concluded first that the amount of dissolved/degraded ZIF-8 is independent of crystallite size and preparation method. However, the authors reported that the amount of dissolved MOF material depended on the ZIF-8/water weight ratio and contact time, with higher ratios yielding lower amounts of dissolved material. The observed inconsistencies in literature for this material were, thus, attributed to differences in sample collection methods.

Although this work did not focus only on the exposure of ZIF-8 in water/moisture, rather also acidic (pH 2 and pH 4) and alkaline environments (pH 11), considering the ZIF-8/solution ratio in further works could be also highly relevant, especially considering the low thicknesses of SURMOF films. 

### 3.3. UiO-66-NH_2_ SURMOF Films

UiO-66 based MOF materials have become a focus point of research over the last years owing to their excelling stabilities in various media. These beneficial properties are commonly attributed to the strength of its carboxylate-Zr^4+^ coordination bonds, as well as its high degree of topological connectivity [46]. Nonetheless, it is important to consider that different functionalizations of the organic linkers can result in changes of stability, as well as complications during synthesis resulting in high defect densities [47]. Below, we will present results which allow to demonstrate that the stability of the UiO-66-NH_2_ SURMOF thin films depends strongly on the pH value; different behaviors are seen in acidic and alkaline environments.

**Figure 3:**
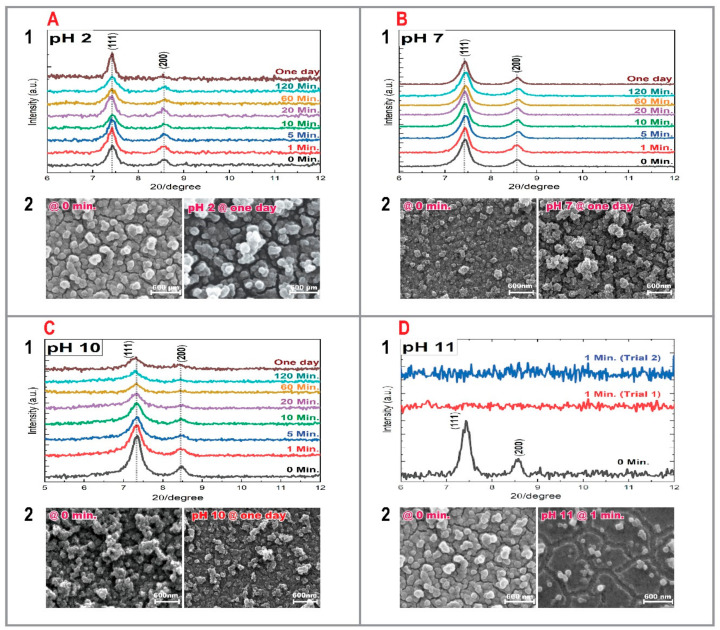
XRD patterns and SEM images of UiO-66-NH_2_ SURMOF films immersed in a: (**A**) pH 2 solution, (**B**) pH 7 solution, (**C**) pH 10 solution, and (**D**) pH 11 solution.

As shown in Figure 3A,B, UiO-66-NH_2_ SURMOFs immersed in an acidic (pH 2) and neutral (pH 7) medium revealed a remarkable stability, with no substantial changes in diffraction peak intensity even after one-day immersion. The morphology of the crystals also showed no significant change. To further study and determine the stability limits of UiO-66-NH_2_, an additional sample was treated in a pH 10 environment as shown in Figure 3C. Under such conditions, the sample’s crystallinity was preserved even after 24 h, although a small decrease in the sum of intensity of the characteristic reflexes could be observed when comparing the as-synthesized sample (t = 0) to the other experiments (c.f. Figure 3C). In general, after 1 hr immersion no significant change in the crystallinity of the sample was detected. The SEM image recorded after a 24 h immersion revealed no significant changes in morphology, although less material could be observed on the surface. Next, the UiO-66-NH_2_ SURMOF film was immersed in a pH 11 solution. As can be observed in Figure 3D, while the UiO-66-NH_2_ SURMOF films proved to be relatively stable in a pH 10 environment, a significant deterioration of the SURMOF could be observed when treated in an aqueous solution of pH 11. The XRD-data and SEM images revealed a pronounced instability and substantial degradation of the film already after just one minute’s immersion. 

Relating these results to other published literature, Kandiah et al. [47] studied the stability of various powder MOFs based on the UiO-66 structure, including UiO-66-NH_2,_ the material studied in this work. In this previous work, two different scenarios (pH 1 and pH 14 for 2 h at room temperature) were investigated. No changes in the crystallinity of the powder samples were observed when treated in HCl or water. Nonetheless, the sample was completely decomposed when treated in NaOH. Leus et al. [48] on the other hand reported high resistance of the material to acidic (pH 0) and basic (pH 12) conditions, although they did observe some decrease in the Langmuir surface area in both scenarios. Lin et al. [49] reported also high stability of the material in water and in fluoride-containing solution. Continuing with water scenarios, a previous work explored the stability of UiO-66-NH_2_ SURMOF films in boiling water [16]. The results showed that for these MOF thin films the crystallinity and the adhesion to the substrate was unchanged after immersion for one hour. This previous finding is in good agreement with the results obtained for the pH 7 scenario explored in this work conducted at less harsher conditions, i.e., room temperature.

It should be also noted that although preserving the material’s crystallinity is of great importance, a stepwise study of the effect on other properties, e.g., surface area, adsorption capacity, must be also considered in future works. Additionally, although in this work the main objective is the exploration of homogeneous and stable films, it should also be noted that controlled MOF release could actually prove to be a benefit for certain applications, such as electrochemical, optical, electronic, sensing, and separation applications.

Finally, to further support the presented data, the thickness behavior of the three SURMOFs (HKUST-1, ZIF-8, and UiO-66-NH_2_) was studied via cross-sectional SEM imaging (c.f. Figure S2, Supplementary Material) before and after immersion in a pH 2 environment during one minute. As can be observed, while the HKUST-1 and ZIF-8 SURMOFs were completely removed from the substrates, the UiO-66-NH_2_ SURMOF showed no considerable changes in thickness.

**Figure 4:**
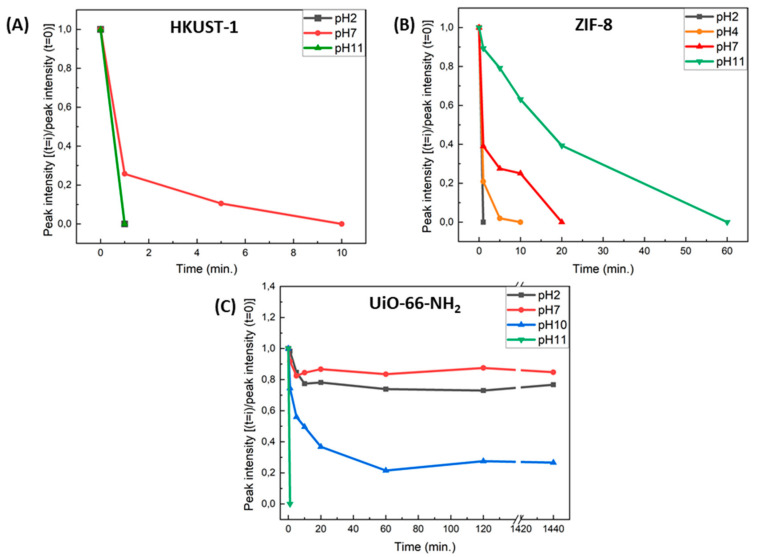
Summary of behavior (XRD peaks) of (**A**) HKUST-1, (**B**) ZIF-8, and (**C**) UiO-66-NH_2_ SURMOFs films as a function of immersion time and pH value.

A summary of all the results obtained for the various SURMOF films with varying pH values is presented in Figure 4.

## 4. Conclusions

The stability of HKUST-1, ZIF-8, and UiO-66-NH_2_ SURMOF films in various acidic and alkaline environments (pH = 2, 4, 7, 10, and 11) was explored and found to strongly vary with immersion times and pH-values. 

The crystallinity of HKUST-1 films was completely lost after immersion in the different solutions (pH 2, pH 7, and pH 11), even in some cases for immersion times as short as 1 min. ZIF-8 SURMOF films were found to be highly unstable in acidic media, particularly below a pH 4 environment, but exhibited overall a stable behavior in alkaline environments when exposed for short periods of time. The highest stability was observed for UiO-66-NH_2_ films, which maintained their highly crystalline structure even after a 24 h immersion period in a highly acidic (pH 2) and neutral environment (pH 7). Furthermore, although the film’s stability was relatively preserved in a pH 10 environment, a pronounced instability was then observed when immersed in a pH 11 basic solution. 

It is very important to consider in future works that although MOF materials may preserve their crystallinity and morphology, it is also crucial to investigate effects in the material’s performance according to its application of interest, e.g., adsorption behavior, surface area, etc.; optimization of the SURMOF films’ quality could be explored in parallel particularly for ZIF-8 in basic environments and UiO-66-NH_2_ in acidic and slightly basic conditions. This work is, therefore, the initial step towards thoroughly understanding the stability of various SURMOF films in acidic and alkaline environments.

## Data Availability

Not applicable.

## Supplementary Materials

The following are available online at https://www.mdpi.com/2077-0375/11/3/207/s1, Figure S1: IRRAS spectra of as-synthesized (A) HKUST-1, (B) ZIF-8, and (C) UiO-66-NH_2_ SURMOF films, Figure S2: SEM cross sectional images of HKUST-1, ZIF-8, and UiO-66-NH_2_ SURMOF films before and after 1 min immersion in a pH 2 solution, Figure S3: Photograph of UiO-66-NH_2_ SURMOF film@gold coated substrate, Table S1: (A) HKUST-1 SURMOF; (B) ZIF-8 SURMOF; (C) UiO-66-NH_2_ SURMOF peaks assign.

## Abbreviations

MOF	metal-organic framework
SURMOF	surface-supported metal-organic framework
LPE	liquid phase epitaxy
LBL	layer-by-layer
SAM	self-assembled monolayer
MHDA	16-mercaptohexadecanoic acid
MUD	11-mercapto-1-undecanol
XRD	X-ray diffraction
SEM	scanning electron microscopy
FEG	Field Emission Gun
IRRAS	infrared reflection absorption spectroscopy
MCT	mercury cadmium telluride
