# Supplementary material for: Stability of Monolithic MOF Thin Films in Acidic and Alkaline Aqueous Media
Source: Membranes (Basel). 2021 Mar 15;11(3):207. doi: 10.3390/membranes11030207 (PMC8001905; doi:10.3390/membranes11030207)
Supplement: Supplementary file 1 [file membranes-11-00207-s001.pdf]

membranes

Supplementary Material: Stability of Monolithic MOF Thin Films in Acidic and Alkaline Aqueous Media

Tawheed Hashem, Elvia P. Valadez Sanchez, Evgenia Bogdanova, Anna Ugodchikova, Alaa Mohamed, Matthias Schwotzer, Mohamed H. Alkordi and Christof Wöll

Note: The substrates used in this work were Au-coated silicon wafers with a 1 mm thickness (100 nm Au thickness on the surface). These substrates were then treated as specified in the experimental section. For the immersion experiments, the samples were then cut into 1 cm × 1 cm pieces.

1. Infrared reflection absorption spectroscopy (IRRAS) Spectra of as-synthesized Surface-Anchored Metal-Organic Framework (SURMOF) samples

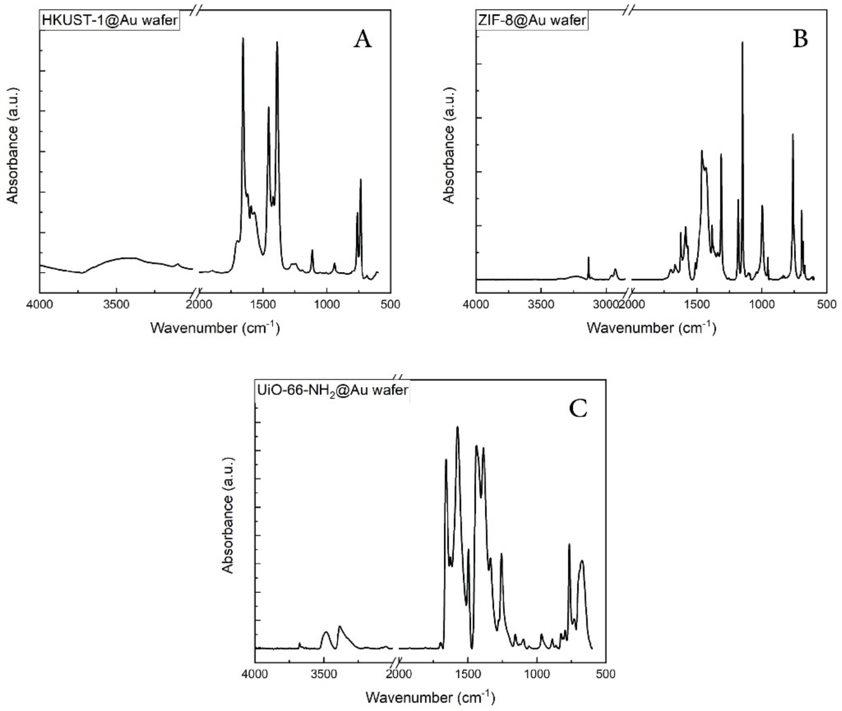

Figure S1. IRRAS spectra of as-synthesized (**A**) HKUST-1, (**B**) ZIF-8, and (**C**) UiO-66-NH$_2$ SURMOF films.

Table S1. (**A**) HKUST-1 SURMOF; (**B**) ZIF-8 SURMOF; (**C**) UiO-66-NH$_2$ SURMOF peak assign.

(**A**)

Copyright: © 2021 by the authors. Licensee MDPI, Basel, Switzerland. This article is an open access article distributed under the terms and conditions of the Creative Commons Attribution (CC BY) license (http://creativecommons.org/licenses/by/4.0/).

Wavenumber (cm^{-1})	F. group	References
**1656 and 1385**	COO^- groups bound to metal ions	Phys. Chem. Chem. Phys., 2010, 12, 8092–8097 [1]<br>ACS Appl. Mater. Interfaces 2017, 9, 7259–7264 [2]

**1455**	Symmetric stretching vibrations of carboxylate groups in BTC	CARBON 49 (2011) 563–572 [3]
**1112 and 1030**	Phenyl groups	Phys. Chem. Chem. Phys., 2010, 12, 8092–8097 [1]
**759 and 735**	Phenyl groups	Phys. Chem. Chem. Phys., 2010, 12, 8092–8097 [1]

(**B**)

Wavenumber (cm⁻¹)	F. group	References
**3135**	Aromatic C-H stretching (imidazole)	Journal of Membrane Science, 361 (2010) 28–37 [4]
**2929**	Aliphatic C-H stretching (imidazole)	Journal of Membrane Science, 361 (2010) 28–37 [4]
**1584**	C=N stretching	Journal of Membrane Science, 361 (2010) 28–37 [4] Journal of Membrane Science 532 (2017) 9–19 [5]
**1384**	CH_3 bending	Journal of Membrane Science 532 (2017) 9–19 [5]
**1100-1400**	C-N absorption bands	Journal of Membrane Science, 361 (2010) 28–37 [4]

(**C**)

Wavenumber (cm⁻¹)	F. group	References
**3477**	Asymmetric vibrations of N–H bonding in the amino group	J. Mater. Chem. A, 2017, 5, 8385–8393 [6]
**3363**	Symmetric vibrations of N–H bonding in the amino group	J. Mater. Chem. A, 2017, 5, 8385–8393 [6]
**1655**	C=C from coordinated carboxylate moieties	J. Mater. Chem. A, 2017, 5, 8385–8393 [6]
**1575**	C=O stretching (from linker)	Ind. Eng. Chem. Res. 2018, 57, 9151−9161 [7]
**1502**	C=C stretching	Ind. Eng. Chem. Res. 2018, 57, 9151−9161 [7]
**1433**	C=C from aromatic	J. Mater. Chem. A, 2017, 5, 8385–8393 [6]
**1256**	C-N bending	Ind. Eng. Chem. Res. 2018, 57, 9151−9161 [7]

2. SURMOF cross-sectional scanning electron microscope (SEM) imaging

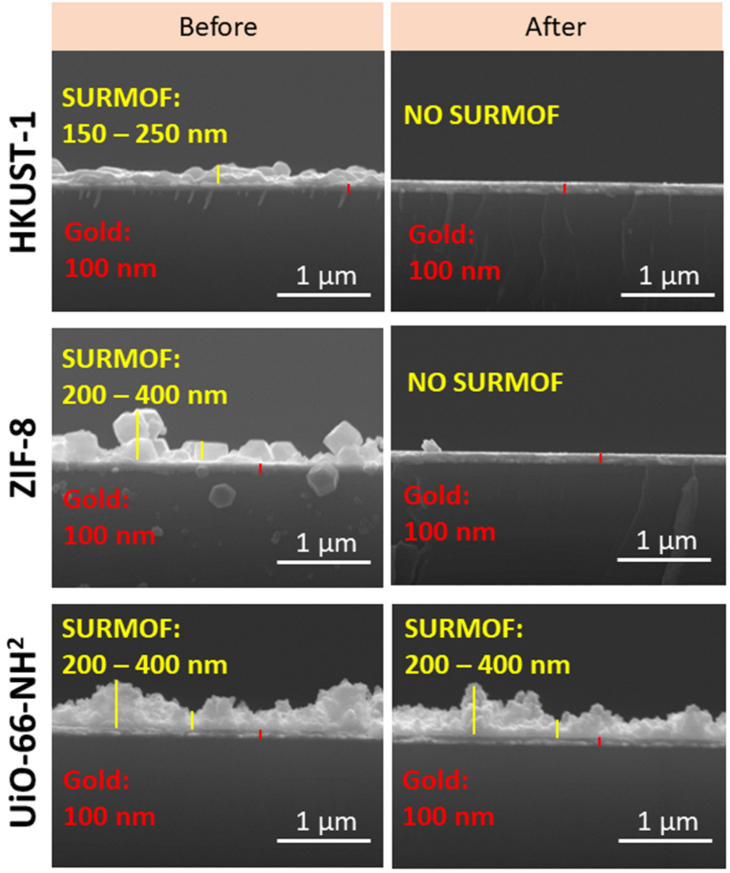

Figure S2. SEM cross sectional images of HKUST-1, ZIF-8, and UiO-66-NH_2 SURMOF films before and after 1 minute immersion in a pH 2 solution.

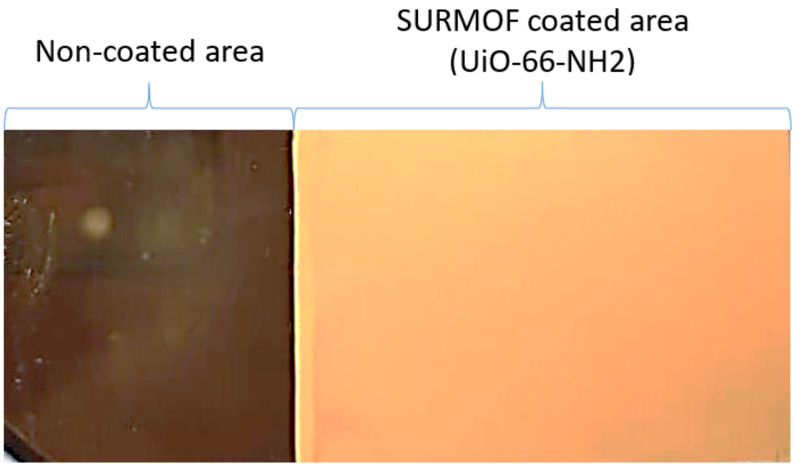

Figure S3. Photograph of UiO-66-NH_2 SURMOF film@gold coated substrate.

References

1. Zybaylo, O.; Shekhah, O.; Wang, H.; Tafipolsky, M.; Schmid, R.; Johannsmann, D.; Wöll, C. A novel method to measure diffusion coefficients in porous metal–organic frameworks. *Phys. Chem. Chem. Phys.* **2010**, *12*, 8092–8098, doi:10.1039/b927601g.

2. Gu, Z.-G.; Chen, S.-C.; Fu, W.-Q.; Zheng, Q.; Zhang, J. Epitaxial Growth of MOF Thin Film for Modifying the Dielectric Layer in Organic Field-Effect Transistors. *ACS Appl. Mater. Interfaces* **2017**, *9*, 7259–7264, doi:10.1021/acsami.6b14541.
3. Petit, C.; Burress, J.; Bandosz, T.J. The synthesis and characterization of copper-based metal–organic framework/graphite oxide composites. *Carbon* **2011**, *49*, 563–572, doi:10.1016/j.carbon.2010.09.059.
4. Ordoñez, M.J.C.; Balkus, K.J.; Ferraris, J.P.; Musselman, I.H. Molecular sieving realized with ZIF-8/Matrimid® mixed-matrix membranes. *J. Membr. Sci.* **2010**, *361*, 28–37, doi:10.1016/j.memsci.2010.06.017.
5. James, J.B.; Lin, Y. Thermal stability of ZIF-8 membranes for gas separations. *J. Membr. Sci.* **2017**, *532*, 9–19, doi:10.1016/j.memsci.2017.02.017.
6. Wang, Y.; Wang, L.; Huang, W.; Zhang, T.; Hu, X.; Perman, J.A.; Ma, S. A metal–organic framework and conducting polymer based electrochemical sensor for high performance cadmium ion detection. *J. Mater. Chem. A* **2017**, *5*, 8385–8393, doi:10.1039/c7ta01066d.
7. Bunge, M.A.; Davis, A.B.; West, K.N.; West, C.W.; Glover, T.G. Synthesis and Characterization of UiO-66-NH2 Metal–Organic Framework Cotton Composite Textiles. *Ind. Eng. Chem. Res.* **2018**, *57*, 9151–9161, doi:10.1021/acs.iecr.8b01010.
